# Role of Gut Microbiota in Pulmonary Arterial Hypertension

**DOI:** 10.3389/fcimb.2022.812303

**Published:** 2022-05-06

**Authors:** Panyun Wu, Tengteng Zhu, Zhen Tan, Shenglan Chen, Zhenfei Fang

**Affiliations:** Department of Cardiovascular Medicine, The Second Xiangya Hospital, Central South University, Changsha, China

**Keywords:** gut dysbiosis, pulmonary arterial hypertension, vascular remodeling, inflammation, treatment

## Abstract

Gut microbiota and its metabolites play an important role in maintaining host homeostasis. Pulmonary arterial hypertension (PAH) is a malignant clinical syndrome with a frightening mortality. Pulmonary vascular remodeling is an important feature of PAH, and its pathogenesis is not well established. With the progress of studies on intestinal microbes in different disease, cumulative evidence indicates that gut microbiota plays a major role in PAH pathophysiology. In this review, we will systematically summarize translational and preclinical data on the correlation between gut dysbiosis and PAH and investigate the role of gut dysbiosis in the causation of PAH. Then, we point out the potential significance of gut dysbiosis in the diagnosis and treatment of PAH as well as several problems that remain to be resolved in the field of gut dysbiosis and PAH. All of this knowledge of gut microbiome might pave the way for the extension of novel pathophysiological mechanisms, diagnosis, and targeted therapies for PAH.

## Highlights

1. It is an indisputable fact that intestinal dysbiosis exists in patients with PAH and rodent models.
2. Gut dysbiosis plays an important role in the pathophysiology of PAH by mediating systemic inflammation or immunity *via* bacteria-related metabolites.
3. Abnormalities in intestinal microflora composition and metabolites contribute to the diagnosis of PAH, and ameliorating intestinal dysbiosis and subsequent disorders are beneficial to the control of PAH, which provides a potentially therapeutic target for PAH.

## Introduction

Pulmonary arterial hypertension (PAH) is a malignant pulmonary vascular disease characterized by pulmonary vascular remodeling, resulting in right heart failure (RHF) and imposing an enormous economic burden on the society (Thenappan et al., 2018; Humbert et al., 2019). Although targeted drugs significantly improved patients’ quality of life and survival by dilating pulmonary arterials, PAH cannot be completely cured. Gut microbiota can synthesize and secrete metabolites, which is critical for maintaining general homeostasis, and, accordingly, gut dysbiosis is associated with the initiation and development of a variety of diseases, such as obesity (Gordon et al., 2006; Henao-Mejia et al., 2012), diabetes (Cani et al., 2008; Khan et al., 2014), atherosclerosis (Brown and Hazen, 2018), hypertension (Marques et al., 2018; Sharma et al., 2019), and heart failure (HF) (Tang et al., 2019). Benefiting from the development of metagenomics, metabonomics, and microbiology, the research studies of gut microbiota and PAH provide a new perspective for elucidating the pathogenesis of PAH. In this review, we discuss the current literature and perspective on the role of gut microbiota in PAH.

## Alteration of Gut Microbiota and Metabolites in PAH: Pre-Clinical and Translational Evidence

PAH is a complex systemic disease involving multiple organs such as the lung, gut, and brain (Oliveira et al., 2020). Profound understanding of the cellular (Archer et al., 2008; Caruso et al., 2017; Zhang et al., 2017), genetic (Drake et al., 2013; Long et al., 2015; Evans et al., 2016), and epigenetic (Rexhaj et al., 2011; Maresca, 2015; Momparler et al., 2017) changes implicated in pulmonary vascular remodeling in patients with PAH has been proved over the past decades (Thenappan et al., 2018; Humbert et al., 2019). Although the precise molecular mechanism of gut microbiota in PAH has not been completely identified, cumulative preclinical and clinical evidence highlights the participation of gut microbiota and its metabolites in the pathogenesis of PAH.

### Gut Dysbiosis in PAH: Pre-Clinical Evidence

Numerous studies have identified that abnormal alterations in gut microbial communities are present both in patients and various animal models with PAH (**Table 1**). The imbalance of the ratio of Firmicutes to Bacteroidetes (F/B), an important characteristic of gut dysbiosis, has been reported in a variety of diseases, such as hypertension (Yang et al., 2015; Marques et al., 2017), HF (Mayerhofer et al., 2020), and obesity (Murphy et al., 2013; Chang et al., 2015). Callejo et al. analyzed the microbial composition of feces in Su5416/Hypoxia (Su/Hx)–treated Wistar rats by 16S rRNA gene sequencing and bioinformatics analysis and observed that the F/B ratio increased by three times compared with control rats, which was mainly related to the decrease of *Bacteroidetes* (Callejo et al., 2018). At the same time, the researchers demonstrated that both acetate-producing bacteria and the level of serum acetate were decreased in PAH rats (Callejo et al., 2018). Similarly, the fecal F/B ratio of Su/Hx-treated SD rats was also significantly higher than that of control and simple hypoxia group. Compared with the control group, the abundance of 14 bacterial genera (e.g., *Rosia* and *Prevotellaceae*) and seven bacterial genera (e.g., *Bacteroides* and *Akkermansia*) in Su/Hx rats increased and decreased, respectively (Sanada et al., 2020). In addition to Su/Hx rat model, Sharma et al. noted that the F/B ratio and pathogenic microorganisms in the fecal of Monocrotaline (MCT)–treated SD rats were profoundly higher than those in the healthy control group, whereas beneficial symbiotic bacteria were richer in healthy control group (Sharma et al., 2020a). Recently, Hong and colleagues found that MCT significantly reduced the microbial diversity and altered the abundance of intestinal flora in Wistar rats, for example, the abundance of *Firmicutes*, *Proteobacteria*, *Actinobacteria*, *Firmicutes-Clostridia*, and *Gammaproteo bacteria* was increased in the PAH group, whereas the abundance of *Bacteroidota*, *Spirochaetota*, *Bacilli*, *Bacteroidia*, and *Spirochaetia* was lower in PAH group than in the control group (Hong et al., 2021). Interestingly, Wedgwood et al. also demonstrated intestinal dysbiosis in the cecum and distal small intestine in postnatal growth restriction (PNGR)–induced PAH rats, including the difference of α/β diversity, the increase in *Enterobacteriaceae*, and decrease in *Lactobacillaceae (* Wedgwood et al., 2020a; Wedgwood et al., 2020b). In addition, a distinct characteristic of microbial community was also presented in C57BL/6 mice subjected to hypoxia, namely, increased α-diversity and decreased F/B ratio, where the genera *Prevotella*, *Oscillospira*, and *Ruminococcus* were increased and *Lactobacillus* was decreased, which was associated with intestinal pathology (Sharma et al., 2020b). Notably, although a few results are controversial, different PAH animal models have similar changes in gut microbiota, namely, an increase in the abundance of pathogenic microorganisms and a decrease in probiotics, suggesting the specific alteration of gut microbiota profile inextricably linked with PAH.

**Table 1:** Contemporary studies on pulmonary arterial hypertension and alterations in gut microbiota.

Article (Date)	Study Population	Summary of Results in PAH	Reference
Callejo et al. (2018)	Su/Hx-treated Wistar rats	increased (↑) Firmicutes/Bacteroides ratio,decreased (↓) Bacteroidetes	(Callejo et al., 2018)
		↓SCFA-producing bacteria: *Parabacteroides*, *Butyricimonas*, *Butyrivibrio*, *Odoribacter*	
		↓serum acetate	
Sanada et al. (2020)	Su/Hx PAH SD rats with or without antibiotics treatment	↑Firmicutes/Bacteroides ratio	(Sanada et al., 2020)
		↑14 bacterial genera: *Rothia*, *Prevotellaceae*, *Parabacteroides*, *Parasutterella*, *Allobaculum*, *Parvibacter*, *Faecalibaculum*, *Ruminococcaceae*, *Bifidobacterium*, *Lachnospiraceae*, *Eubacterium coprostanoligenes group, Coprococcus 3*, *Acetitomaculum*.	
		↓7 bacterial: *Bacteroides*, *Akkermansia*, *Dehalobacterium*, *Marvinbryantia*, *Enterococcus*, *Bacteroidetes S24-7 group uncultured bacterium*.	
		Antibiotic treatment: relieves the vascular remodeling, RVH, and RVSP in Su/Hx rats.	
Sharma et al. (2020)	MCT or chronic angiotensin II–treated SD rats	↑Firmicutes/Bacteroides ratio	(Sharma et al., 2020a)
		↑*Corynebacterium*, Osc*illospira*, *Roseburia*, *Akkermansia*, *Clostridiales*, *Aerococcaceae*	
		↓SCFA-producing bacteria: *Bifidobacterium* and *Streptococcus*	
		↑sympathetic nervous activity	
		↑I-FABP, TIMP-1, HMGB1	
Hong et al. (2021)	MCT treated Wistar rats	↓Alpha-diversity	(Hong et al., 2021)
		↑*Firmicutes*, *Proteobacteria*, and *Actinobacteria*, *Firmicutes-Clostridia*, *Gammaproteo bacteria*, *Allobaculum*, *Ralstonia*, *Bifidobacterium*, *Turicibacter*, *Candidatus_Saccharimonas*, and *Clostridium_sensu_stricto_1*	
		↓*Bacteroidota*, *Spirochaetota*, *Bacilli*, *Bacteroidia*, *Spirochaetia*, *Lactobacillus*, *Romboutsia*,	
Wedgwood et al. (2020)	PNGR with or without hyperoxia and probiotic *Lactobacillus reuteri* DSM 17938 or TLR4 inhibitor	↓Lactobacillaceae	(Wedgwood et al., 2020a; Wedgwood et al., 2020b)
		↑intestinal Enterobacteriaceae	
		↑IL1β; ↓IκBα, NFκB	
		DSM 17938 treatment: ↓alpha-diversity and *Proteobacteria*, attenuated PH and RVH in pups with PNGR	
		TLR4 inhibitor TAK-242: attenuated PH and inflammation	
Sharma et al. (2020)	ACE2 knockin and wild-type C57BL/6 mice with or without chronic hypoxia	↓Firmicutes/Bacteroides ratio, ↓*Firmicutes*, ↑*Bacteroidetes*	(Sharma et al., 2020a)
		↑Alpha-diversity;	
		↑phylum *Proteobacteria*, *Prevotella*, *Oscillospira* and *Ruminococcus*	
		↓*Lactobacillus*	
		FMT from ACE2 knockin mice: attenuated hypoxia-induced PAH, gut pathology, gut dysbiosis, increase RVSP and RVH	
Kim et al. (2020)	PAH patients	↓alpha-diversity	(Kim et al., 2020)
		↑TMA/TMAO-producing taxa: *Clostridium*, *Prevotella*, *aerofaciens*, *Clostridium*, *Staphylococcus*, *Streptococcus*, *Citrobacter*, and *Collinsella*, et al.	
		↓butyrate-and propionate-producing bacteria: *Coprococcus*, *Butyrivibrio*, *Lachnospiraceae*, *Eubacterium*, *Akkermansia*, *and Bacteroides*, *et al.*	
		↑arginine, proline, ornithine, purine and urate metabolism: such as xanthine oxidase and purine nucleosidase	
Goel et al. (2017)	PAH patients	↓alpha-diversity	(Goel et al., 2017)
		↑plasma zonulin, iFABP, LPS, HMGB1	
Zhang et al. (2020)	PH patients	↑microbiota richness; ↓the community diversity	(Zhang et al., 2020)
		↑*Firmicutes*, *Unclassified_k_norank*, *Chloroflexi*; *Streptococcaceae*, *Leptotrichiaceae; Leptotrichia*, *Streptococcus*, *Lautropia*, and *Ralstonia*	
		↓*Bacteroidetes*, *Saccharibacteria*, *SR1_Absconditabacteria*; *Porphyromonadaceae*, *Flavobacteriaceae*; *Carnobacteriaceae*, *Granulicatella*, and *Alloprevotella*	
Huang et al. (2022)	IPAH patients MCT-treated SD rats	↑microbial TMA-generating enzyme CutC, TMAO, IL6, CXCL1, CXCL2, and CXCL6	(Huang et al., 2022)

### Gut Dysbiosis in PAH: Translational Clinical Evidence

In addition to PAH animal models, a growing body evidence confirmed the presence of gut microbial dysbiosis in patients with PAH (Goel et al., 2017; Kim et al., 2020; Zhang et al., 2020). Goel et al. demonstrated that the abundance, diversity, and evenness of gut microbiota in patients with PAH were profoundly decreased, whereas Gram-positive and facultative-anaerobic genera were increased. Seungbum et al. accomplished a landmark study in the field of gut microbiota and PAH *via* shotgun metagenomics, which firstly observed a unique profile of gut microbial communities in patients with PAH (Kim et al., 2020). Fecal analysis of 18 patients with type 1 PAH and 13 reference subjects revealed that PAH-associated flora involving in the synthesis of arginine, proline, and ornithine, as well as colonies related to trimethylamine/trimethylamine N-oxide (TMA/TMAO) and purine metabolism were increased in PAH cohorts, whereas the abundance of butyric- and propionate-producing bacteria such as *Coprococcus* and *Butyrivibrio* were richer in health controls. This study noted that the distinct gut microbiome was effective in predicting PAH with 83% accuracy. Furthermore, virome analysis confirmed *Enterococcal* enrichment and relative depletion of *Lactococcal* phages in patients with PAH (Kim et al., 2020). In addition, Zhang and colleagues studied the microbiota profile of oropharyngeal rather than fecal samples in patients with pulmonary hypertension (PH), and the results indicated that the relative abundance and diversity of microbiota in patients with PH were profoundly different from healthy subjects, including that *Streptococcus*, *Lautropia*, and *Ralstonia* were enriched in PH. Although *Saemophilus*, *Rothia*, *Granulicatella*, *Capnocytophage*, and *Sccharibacteria* were relatively abundant in healthy subjects, this difference may be a potential predictor for distinguishing PH from reference subjects (Zhang et al., 2020). Although limited by sample size and geographic area, this study was partly consistent with preclinical studies and reflected the characteristics of gut microbiota composition alteration in PAH. In addition, future randomized controlled studies with larger sample sizes await further confirm these findings.

### Gut Microbial Metabolites in PAH

Intestinal microflora can synthesize and secrete specific metabolites, which play a vital role in regulating various host physiological function. Given that, the alteration in serum levels of these metabolites may reflect the taxonomic and functional changes of gut microbiota and diversity in PAH. For example, Callejo and colleagues measured serum concentrations of short-chain fatty acids (SCFAs) in Su/Hx PAH rats by nuclear magnetic resonance spectroscopy and found a significant decrease in acetate, which was consistent with the decrease in acetate-producing bacteria (Callejo et al., 2018). Serum endotoxin, another important metabolite of gut microbiota (Kazemian et al., 2020), was involved in PAH pathophysiology by inducing inflammation and subsequently pulmonary vascular remodeling (Thenappan et al., 2011). Common bile duct ligation in rats resulted in elevated serum endotoxin levels and pulmonary vascular disease (Thenappan et al., 2011; Ranchoux et al., 2017). More directly, increased serum endotoxin concentration in the portal vein was also observed in MCT-PAH rats (Ranchoux et al., 2017). These studies suggest that changes of the gut microbial metabolites arising from gut dysbiosis may contribute to the initiation, maintenance, or aggravation of pulmonary vascular adverse remodeling in PAH.

## The Causal Relationship Between Gut Microbiota and PAH

**Figure 1:**
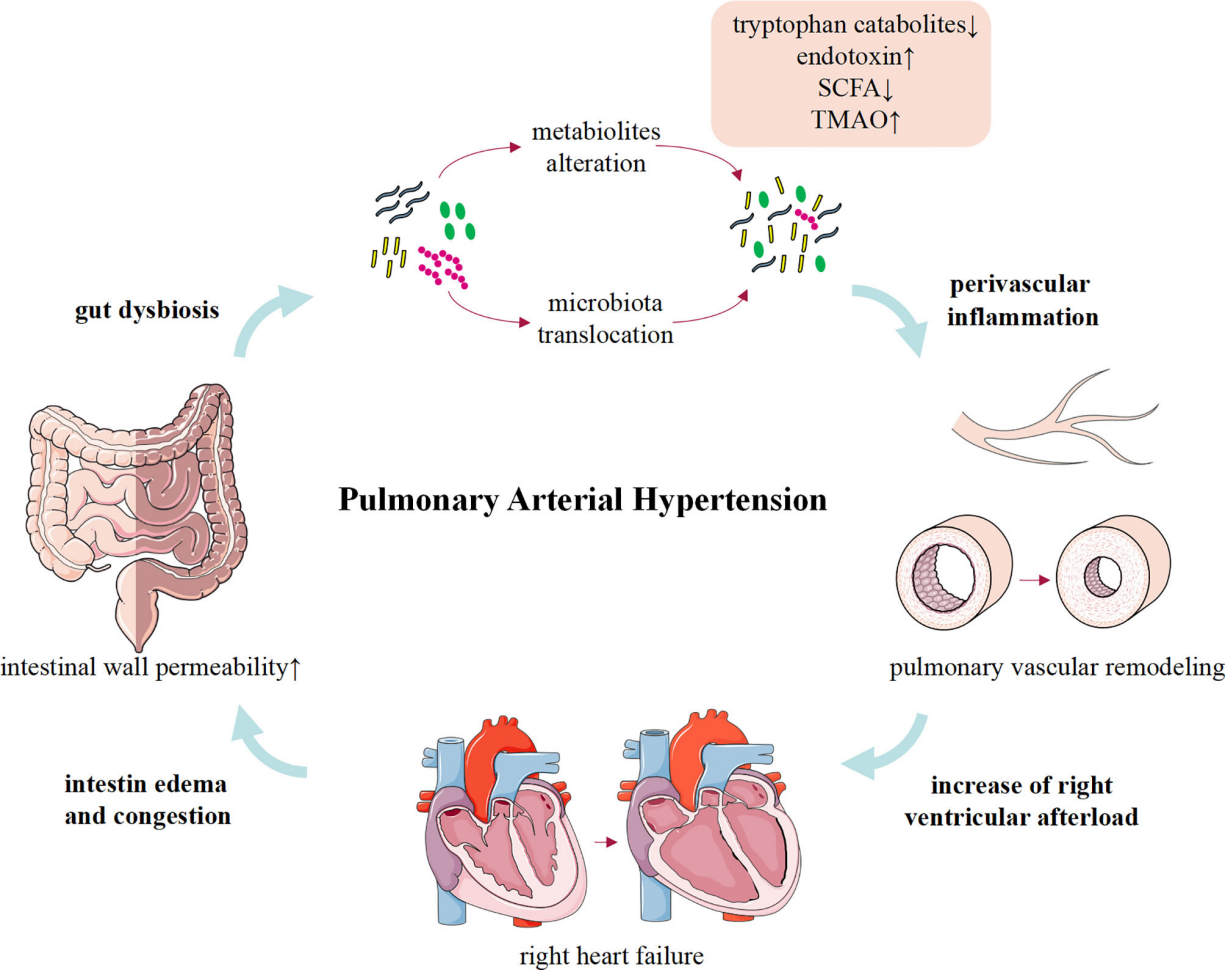
The role and potential mechanism of gut dysbiosis in pulmonary arterial hypertension.

Although limited to rare human studies, convincing animal data linking gut dysbiosis to PAH have been available (**Figure 1**). Gut microbiota can modulate the host’s immune condition, and alterations of gut microbiota composition and the activation of inflammation support that gut dysbiosis involves in the inflammation process of PAH (Rabinovitch et al., 2014; Marsh et al., 2018; Sweatt et al., 2019). Meanwhile, PAH is a complex clinical syndrome and gut dysbiosis may be a concomitant symptom of RHF or other pathogenic factors. Therefore, more comprehensive research studies are urgently required to determine whether the gut dysbiosis is a cause or merely a consequence of PAH.

### Gut Dysbiosis and Inflammation in PAH

Despite lacking in direct evidence to elucidate that gut microbiota contributes to the development of PAH, gut dysbiosis causes pathophysiological changes similar to PAH. Intriguingly, an important feature of pulmonary arterial lesions in patients with PAH and experimental models is varying degrees of perivascular inflammatory infiltration. Although the mechanisms that trigger and/or aggravate the inflammatory response are not fully understood, inflammation precedes vascular lesion supports that immune dysregulation is responsible for vascular remodeling in PAH (Tamosiuniene et al., 2011). Increasing evidence suggested that a healthy gut microbial community contributes to maintaining the host immune homeostasis (Kim et al., 2016; Clavel et al., 2017), and gut dysbiosis is associated with a variety of inflammation-related diseases, including atherosclerosis (Jonsson and Bäckhed, 2017; Yoshida et al., 2018; Wu et al., 2021), obesity (Yoshimoto et al., 2013; Canfora et al., 2019; Arnoriaga-Rodríguez et al., 2020), multiple sclerosis (Kadowaki et al., 2019; Kadowaki and Quintana, 2020; Pröbstel et al., 2020), chronic transplantation rejection (Wu et al., 2020), autoimmune diseases (Manfredo Vieira et al., 2018; Fine et al., 2020; McPherson et al., 2021; Pandey et al., 2021), and cancers (Zhu et al., 2020; McPherson et al., 2021). Considering that inflammation is a crucial factor in the initiation and progress of PAH (Almodovar et al., 2011; Stacher et al., 2012; Graham et al., 2013; Simonneau et al., 2013; Saito et al., 2017; Qian et al., 2019). Recently, accumulating animal and clinical evidence reveals that gut dysbiosis may play an important role in inducing or aggravating the inflammatory response in PAH *via* the following aspects. First, gut dysbiosis disrupts gut barrier function and migrates gut microbiota into the host circulation triggering the immunoreaction (Manfredo Vieira et al., 2018; Fine et al., 2020). Second, gut microbiota participates in the regulation of host immune response by governing the metabolism of endotoxin, SCFAs, tryptophan, and TMA/TMAO (Thenappan et al., 2019).

The intestinal mucosal barrier maintains homeostasis by preventing pathogenic microorganisms or toxins from entering other tissues, organs, or blood (Citi, 2018). A healthy gut microbial community is beneficial to maintain normal intestinal barrier, whereas gut dysbiosis impaired the integrity of barrier function (Castillo et al., 2019). Intestinal pathological examination in MCT-PAH rats showed significantly increased intestinal fibrosis and muscular thickness, decreased goblet cells, and shortened villus length. Meanwhile, plasma intestinal fatty acid binding protein (I-FABP) in MCT-PAH rats increased by 2.3 times compared with healthy rats (Sharma et al., 2020a). This intriguing study firstly identified that MCT-PAH is associated with impairment of intestinal barrier function and increased intestinal permeability. All these changes significantly affect the host–microbiota interaction, which may trigger a host immune response and destroy the homeostasis of the gut-lung axis. Furthermore, these pathological changes may also provide a suitable growth environment for pathogenic microbiota and produce a variety of pro-inflammatory substances, consequently affecting the development of PAH. However, these data need to be validated in more patients with PAH and animal models in the future.

With the alteration of gut microbial composition and the increase of intestinal permeability, endotoxins translocate into the host circulation through the intestinal wall and cause metabolic endotoxemia (Bowser et al., 2020). Previous studies demonstrated that serum endotoxin and sCD14 levels were significantly increased in patients with PAH and MCT-treated rats (Ranchoux et al., 2017), which promoted immune response and pulmonary vascular remodeling *via* activation of the Toll-like Receptor 4 (TLR4)/nuclear factor kB (NF-kB) inflammatory signaling pathway and confirmed intestinal bacterial translocation and macrophage activation in PAH (Perros et al., 2011; Bauer et al., 2014; Ranchoux et al., 2017). Accordingly, depleted macrophages or targeted therapy improved bacterial translocation and pulmonary vascular remodeling (Thenappan et al., 2011; Ranchoux et al., 2017). These studies suggest that gut dysbiosis, impaired barrier function, and elevated circulating endotoxin levels may be important pathways that trigger the inflammatory response of PAH.

Gut microbiota produce a variety of metabolites, and their role in regulating host metabolic balance has been confirmed in past descades (Tilg et al., 2020). SCFAs (such as acetate, propionate, and butyrate), tryptophan metabolites, TMA/TMAO, and bile acids involved in the pathogenesis of PAH have been extensively studied. SCFAs, the end products of microbial fermentation, exert anti-inflammatory immunomodulatory effects by activating G protein–coupled receptor or inhibiting histone deacetylase (Kaisar et al., 2017; Ratajczak et al., 2019; Zhan et al., 2019). Previous studies demonstrated that SCFA-producing bacteria and SCFAs were significantly reduced in patients with PAH and rodent models (Callejo et al., 2018; Kim et al., 2020). Although direct data remain scarce, these results suggest that gut dysbiosis leads to the reduction of SCFAs and exacerbates the inflammatory response and PAH.

Indole, a tryptophan catabolite synthesized by intestinal bacterial possessing tryptophanase, was also reported to play important roles in inflammation and immune tolerance *via* inhibiting TNF-αmediated NF-κB activation, expression of IL-8, and increasing expression of the anti-inflammatory IL-10 (Bansal et al., 2010). In addition, indole also plays a beneficial effect on increasing the expression of epithelial junction complex molecules and on improving intestinal epithelial cell and barrier function (Bansal et al., 2010; Shimada et al., 2013). It is intriguing that indole is produced by various commensal Gram-positive and Gram-negative bacteria (Lee and Lee, 2010; Lee et al., 2015), and the amount of several indole-producing bacteria, such as *Escherichia coli (* Lee et al., 2007), *Lactobacillus strain (* Natividad et al., 2018; Sharma et al., 2020b), *Prevotella (* Sanada et al., 2020; Sharma et al., 2020b), and *Bacteroidetes (* Callejo et al., 2018; Sharma et al., 2020a), is extremely abnormal in patients with PAH and rodent models. In view of this, we conclude that the adverse effects of abnormal inflammation and immune response caused by gut dysbiosis in PAH are partially mediated by indoles. Recent study using metagenomic and linear discriminant analysis effect size analysis observed increase in *Streptococcus*, *Coprococcus*, and bacterial tryptophan biosynthesis in patients with PAH (Kim et al., 2020). Previous studies have shown that tryptophan hydroxylase 1 inhibitor can ameliorate pulmonary vascular pathology (MacLean, 2007). These findings suggest gut microbiome affect PAH by regulating the metabolism of tryptophan and inflammation.

TMA is synthesized by gut microbes using choline, carnitine, and other food ingredients and is converted to TMAO in the liver, which contributes to vascular endothelial dysfunction *via* inducing inflammation and oxidative stress (Li T et al., 2017). Previous studies have confirmed that TMAO was actively participated in the development of various vascular disease (Gregory et al., 2015; Senthong et al., 2016; Li XS et al., 2017), and the level is affected by the composition and diversity of gut microbiota. TMA/TMAO-producing bacteria, such as *Clostridium*, *Desulfovibrio*, *Enterobacter*, *Escherichia*, *Klebsiella*, *Pseudomonas*, *Rothia*, *Prevotella*, *Clostridium*, *Staphylococcus*, *Streptococcus*, *Citrobacter*, and *Collinella*, were significantly increased in patients with PAH, which were negatively correlated with TMA/TMAO production (Kim et al., 2020). Intriguingly, circulating TMAO was elevated in severe patients with PAH and MCT-treated rats compared to healthy control and hypoxia-induced mouse models (Huang et al., 2022), and the reason for this difference remains to be further elucidated (Aldred, 2022). In addition, the study also confirmed that TMAO promotes the proliferation and migration of PASMCs *via* upregulating inflammatory factors secretion from macrophages, and reducing the production of TMAO with 3,3-dimethyl-1-butanol (DMB) partially alleviated pulmonary vascular remodeling by reducing inflammatory factors (Huang et al., 2022). This indicates that the inflammatory pathway involved in TMAO may directly contribute to the pathogenesis of PAH.

### Gut Dysbiosis and RHF in PAH

RHF is a serious complication in patients with end-stage PAH and suggests a poor prognosis. Available data indicated that intestinal morphology and permeability were altered in patients with HF (Sandek et al., 2007; Arutyunov et al., 2008). Patients with HF may present with intestinal wall thickening, intestinal wall edema, and impaired barrier function (Sandek et al., 2008). While decreased cardiac output and activation of RAAS in HF results in adaptive redistribution of blood between different organs to satisfy perfusion of vital organs, which further restricts intestinal blood flow. Interestingly, Sandek et al. and Krack et al. demonstrated that restricted intestinal blood flow in patients with HF results in abnormal growth of intestinal bacteria and impaired intestinal barrier function (Krack et al., 2005; Sandek et al., 2008). In addition, previous studies have identified the specific characteristics of intestinal microbiome profile in patients with HF, namely, decreased intestinal microbial diversity (mainly driven by *Blautia* and *Collinsella*) and downregulation of important microflora, as well as the overgrowth of intestinal pathogenic bacteria such as *Campylobacter*, *Shigella*, *Salmonella*, *Yersinia enterocolitica*, and *Candida species* (Pasini et al., 2016; Luedde et al., 2017). Significantly, microbial metabolites such as endotoxin, SCFAs, and TMAO are affected by gut dysbiosis, which were consistent with changes in intestinal microbes and were also observed in PAH animal models and patients (Kim et al., 2020). On the one hand, bacterial translocation and gut dysbiosis lead to persistent low-grade chronic inflammation in patients with HF (Dick and Epelman, 2016; Tang et al., 2017). On the other hand, the toxic effects of microbial metabolites, such as TMAO, may directly lead to cardiac mitochondrial dysfunction, cardiac hypertrophy, and fibrosis (Organ et al., 2016; Makrecka-Kuka et al., 2017; Li et al., 2019). Therefore, gut dysbiosis and HF are mutually progressive “vicious circle”. Together, these changes in intestinal pathology, microbiota, and barrier function facilitate the translocation of bacteria and/or metabolites and subsequently systemic inflammation, which increase susceptibility to HF. In future studies, it is necessary to further clarify the relationship between gut dysbiosis and HF in PAH by matching the pathophysiological status of enrolled subjects.

### Pathogenic Factor and Gut Dysbiosis in PAH

Apart from idiopathic PAH (IPAH), PAH is always associated with other conditions, including infectious disorders [such as human immunodeficiency virus (HIV) (Vujkovic-Cvijin and Somsouk, 2019) and schistosomiasis infection (Jenkins et al., 2018; Floudas et al., 2019; Hu et al., 2020)], portal hypertension (Yokoyama et al., 2020), and connective tissue diseases [systemic lupus erythematosus (SLE) (Luo et al., 2018), multiple sclerosis (Chen et al., 2016), and rheumatoid arthritis (RA) (Lerner and Matthias, 2015; Manasson et al., 2018)], and these primary diseases are usually accompanied with gut dysbiosis. The potential association between the primary disease and gut dysbiosis in PAH needs to be investigated. Rexhaj and colleagues analyzed the gut microbiota of fecal in rats with systemic hypertension and MCT-PAH and found that *Bifidobacterium* and *Streptococcus* were significantly increased in hypertension group, whereas the number of *Spirillum*, *Rosa*, and *akermansia* was increased in MCT-PAH group (Sharma et al., 2020a). It suggested that the composition of gut microbiota in diverse diseases may present unique characteristics. Distinguish disease related unique gut microbiota alteration might be beneficial in identifying types of PAH.

Schistosomiasis-associated PAH (Sch-PAH) is a serious complication of chronic hepatosplenic schistosomiasis, which is related to inflammation and transforming growth factor (TGF) signaling pathway, and histopathologic features are similar to that of IPAH (Lapa et al., 2009; Knafl et al., 2020). Recent studies reported that *Schistosoma mansoni*, the primary etiology of Sch-PAH, could regulate the composition and diversity of intestinal microbiota and immunity (Jenkins et al., 2018; Floudas et al., 2019). Analysis of fecal microbiota of *Schistosoma mansoni–*infected mice revealed that the abundance of *Alistipes*, *Bacteroides*, *Parabacteroides*, and *Helicobacte*r was increased, whereas the abundance of *Lactobacillus* was decreased. In addition to dysbacteria, *Schistosoma mansoni* also altered the metabolic signature of infected mice, including increased glycolysis and decreased microbial-related metabolites (e.g., SCFA), which well known to play important roles in PAH (Wang et al., 2004).

HIV infection increased the risk of a variety of infectious and non-infectious pulmonary conditions (Almodovar et al., 2011) and is a well-established risk factor for PH (Opravil and Sereni, 2008; Hai-long et al., 2013). Aberrant inflammation and immune activation impaired gastrointestinal barrier function, and then, intestinal bacteria and bacterial products were transferred to the systemic circulation and further promote inflammation and disease progression, which plays an important role in morbidity and mortality in patients with HIV (Marchetti et al., 2013; Deeks et al., 2013). Numerous studies have confirmed that a series of changes in the composition of the gut microbiome in HIV-infected patients (Ellis et al., 2011; Marchetti et al., 2013). Gori and colleagues demonstrated that the fecal microbiota of HIV-infected patients had higher levels of opportunistic pathogens (e.g., *P. aeruginosa* and *C. albicans*) and lower levels of protective bacteria (e.g., *lactobacilli* and *bifidobacteria*) compared with healthy individuals (Gori et al., 2008), similar to previous preclinical and clinical studies on PAH. In addition, HIV infection–related hypercoagulable, endothelial damage, and dysfunction also contribute to the development of PAH. However, the pathogenesis of intestinal flora in HIV-associated PH remains to be elucidated constantly and further investigated.

SLE is complex autoimmune diseases. Intestinal microbiota dysbiosis has been observed in patients with SLE and lupus models. Luo and colleagues reported that the composition and diversity of intestinal microorganisms in lupus-prone mice (NZB/W F1) and patients were considerably altered and correlated with disease severity (Luo et al., 2018). In patients with SLE, some Gram-negative bacteria, such as *Proteobacteria*, were significantly increased. Whereas different from reduced F/B ratio in patients with SLE in remission in a previous study (Hevia et al., 2014), no significantly difference was observed in the F/B ratio between patients with and without SLE in this study. In addition, increased bacterial diversity was also confirmed in lupus-prone MRL/LPR and SWRxNZB F1 (SNF1) mouse models, characterized by a decrease in *Lactobacillaceae* and an increase in *Rikenellaceae* family or *Lachnospiraceae*, which was associated with lupus-like symptoms (Zhang et al., 2014; Johnson et al., 2015).

In short, intestinal dysbiosis is manifested in various primary diseases, and correction of intestinal dysbiosis is beneficial to alleviate these diseases. However, intestinal microbiota is easily affected by various factors, such as health status, diet, and drugs. Therefore, it is difficult to determine the contribution of these primary diseases to intestinal dysbiosis, and the role of dysbiosis in PAH progress needs to be further explored.

## Future Prospects of Intestinal Microbiota in PAH

Gut microbiota and its metabolites are closely related to host homeostasis (Fan and Pedersen, 2021). Therefore, identifying specific alteration of gut microbial composition and function may provide potential diagnostic and therapeutic approaches. Recent studies noted that the composition of intestinal flora in PAH rats is significantly different from that of healthy control subjects and systemic hypertension rats (Callejo et al., 2018; Sharma et al., 2020a). In Seungbum’s study, patients with PAH were predicted with 85% accuracy according to the composition of gut microbiota. This evidence suggested that integrating existing studies and dynamically monitoring the composition of gut microbiota in large-scale PAH population is expected to screen out valuable biomarkers for clinical diagnosis based on the changes of gut microbiota. However, its repeatability must be confirmed in future research to improve the stability and feasibility of intestinal flora as a diagnostic marker of PAH.

The accumulating evidence demonstrated that modification of the gut microbiota can suppress the progression of several chronic diseases (Olofsson and Bäckhed, 2022; Schupack et al., 2022), including metabolic diseases (e.g., diabetes, obesity), cardiovascular diseases (e.g., atherosclerosis and hypertension) and tumors. Considering the contribution to PAH, intestinal flora has emerged as a potential therapeutic target for PAH (Sanada et al., 2020). Numerous factors such as age, dietary patterns, drugs, genetics, and environmental microbes have profound effects on host microbiota, and naturally regulating the composition of gut microbiome through diet, antibiotics, probiotics, or fecal microbiota transplantation (FMT) may be an effective option for PAH.

The adverse effects of high-fat diet on intestinal microbiome and metabolites have been demonstrated in a host of diseases, and high fat aggravates pulmonary vascular abnormalities and right ventricular hypertrophy (RVH) in apolipoprotein E–null mice (Lawrie et al., 2011; Wallström et al., 2012). Specific dietary patterns, such as foods rich in dietary fiber and antioxidant food components, play important roles in maintaining gut homeostasis and cardiovascular protection by improving lipid profiles, reducing inflammation, and modulating gut microbiota and its metabolites (e.g., SCFAs) (Gill et al., 2018; Hills et al., 2019; Martin-Gallausiaux et al., 2020; Zhou et al., 2021). For example, the Mediterranean diet was associated with improved health status and predicted future cardiovascular disease risk independently of traditional risk factors (Li et al., 2020). In addition, nutritional alterations such as iron deficiency are prevalent in PAH and may trigger or exacerbate disease progression (Callejo et al., 2020). Available evidence indicates that dietary components such as polyphenols alleviate disease progression in animal models of PAH. However, the effects of dietary nutrition intervention and timing choices targeting intestinal microbiota on PAH remain to be further confirmed (Aldred, 2022).

Antibiotics are prevalent anti-infection drugs in clinical practice that can collaterally alter the gut microbial profiles (Zimmermann et al., 2021). Sanada et al. observed that antibiotic intervention could alter the composition of intestinal flora and alleviated pulmonary vascular remodeling and RVH index in Su/Hx-rats (Sanada et al., 2020). Notably, antibiotics are strongly related to microbial dysbiosis (Blaser, 2016), such as fungal enrichment, and strict clinical indications limit the use of antibiotics in PAH to a certain extent.

Evidence has confirmed that probiotics, living microorganisms, ameliorate certain disease (e.g., irritable bowel syndrome and inflammatory bowel disease) by replenishing depleted host microbes, which provide reference for the treatment of PAH with probiotics (Ford et al., 2014; Olofsson and Bäckhed, 2022). Wedgwood and colleagues demonstrated that intervention with *Lactobacillus reuteri* DSM 17938 in PNGR rats reduced α-diversity of gut microbiota and prevented PNGR-associated PH and RVH (Wedgwood et al., 2020a). Sharma et al. first confirmed that overexpressed ACE2 prevents intestinal flora and intestinal pathology related to PH, and transplantation of feces of mice with overexpressed ACE2 was beneficial to correct these pathological changes and relieve the increased right ventricular systolic pressure and RVH caused by hypoxia in wild-type mice (Sharma et al., 2020b). The aforementioned studies suggest that probiotics and/or FMT may be a promising complementary therapy for PAH, and the complementarity between donor and recipient of fecal bacteria may further improve the therapeutic efficacy. Recently, Daphne et al. achieved a Food and Drug Administration–approved phase I safety and feasibility trial of microbiota transplantation for PAH, which is the first step to explore FMT as a treatment for patients with PAH. If FMT is both safe and feasible in PAH, then future clinical studies could further examine its efficacy (Moutsoglou, 2022). In addition, gut dysbiosis results in corresponding changes in bacterial metabolites, and previous studies have demonstrated that supplementing exogenous SCFAs (Maniar et al., 2018; Karoor et al., 2021) or reducing TMAO (Huang et al., 2022) can relieve pulmonary vascular disease, suggesting that correction of abnormal metabolites may be another potential therapeutic strategy for PAH.

## Conclusion

PAH is a clinical syndrome involving multiple systems, and accumulating evidence demonstrated that intestinal microbiota plays an important role in PAH. Targeting intestinal microbiota, such as probiotics and FMT, has profoundly therapeutic potential in PAH and may be an essential complement to targeted vasodilation therapies. It is remarkably, however, that the improvement of intestinal microflora in pulmonary vascular remodeling is based primarily on animal experiments, and there is still a long way to go for intestinal microbiota as a therapeutic target in clinical practice. First, the causal relationship between gut dysbiosis and changes in metabolites and PAH awaits further determined. Second, well-designed large-scale clinical studies are necessary to verify the role of intestinal microbiota in PAH. In conclusion, the exploration of intestinal flora and its function provides a new perspective for examining the pathogenesis and treatment strategies of PAH.

## Author Contributions

PW and TZ drafted and contributed equally to this manuscript. ZT, SC, and ZF revised the manuscript. All authors contributed to the article and approved the submitted version.

## Conflict of Interest

The authors declare that the research was conducted in the absence of any commercial or financial relationships that could be construed as a potential conflict of interest.

## Publisher’s Note

All claims expressed in this article are solely those of the authors and do not necessarily represent those of their affiliated organizations, or those of the publisher, the editors and the reviewers. Any product that may be evaluated in this article, or claim that may be made by its manufacturer, is not guaranteed or endorsed by the publisher.
